# Supplementary material for: Defective Viral Genomes Reveal History-dependent Self-regulation of Type I Interferon Dynamics
Source: bioRxiv. 2026 Oct 31:2026.10.30.763053. Preprint. [Version 1] doi: 10.64898/2026.10.30.763053 (PMC13734136; doi:10.64898/2026.10.30.763053)
Supplement: Supplementary file 1 [file NIHPP2026.10.30.763053v1-supplement-1.pdf]

bioRxiv preprint doi: https://doi.org/10.64898/2026.10.30.763053; this version posted October 31, 2026. The copyright holder for this preprint (which was not certified by peer review) is the author/funder, who has granted bioRxiv a license to display the preprint in perpetuity. It is made available under a CC-BY 4.0 International license.

Supporting Information

bioRxiv preprint doi: https://doi.org/10.64898/2026.10.30.763053; this version posted October 31, 2026. The copyright holder for this preprint (which was not certified by peer review) is the author/funder, who has granted bioRxiv a license to display the preprint in perpetuity. It is made available under a CC-BY 4.0 International license.

Table S1. Posterior medians with 90% credible intervals [5%, 95%] for the with-DVG model across inoculum groups. The three groups are fitted jointly: host-related parameters are shared or partially pooled across groups, whereas inoculum- and DVG-related parameters are estimated separately by group, as indicated in the final column. IFN dynamics include induction and cumulative-IFN negative feedback; below-LOD observations are left-censored. Priors for the infected-cell loss rate (δ), virus clearance rate (c), and IFN decay rate (α) were informed by published within-host influenza estimates [33, 34, 40, 71]; all prior distributions and bounds are given in Table S2. In the low- and medium-DVG groups, cumulative IFN exposure remains below the range in which negative feedback is appreciably engaged; accordingly, the data primarily constrain $g(\mathrm{cum}F)$ to remain near one rather than precisely identifying $\mathrm{cum}F_{50}$. The large $\mathrm{cum}F_{50}$ estimates in these groups therefore represent weak feedback over the observed time window rather than precisely estimated thresholds. In the high-DVG group, cumulative IFN exposure enters the feedback-sensitive range, allowing $\mathrm{cum}F_{50}$ to be more directly constrained.

Parameter	LOW	MED	HIGH	Across groups
β	4.83e-10 [1.17e-10, 1.05e-9]	1.16e-10 [5.09e-11, 2.73e-10]	6.98e-10 [2.35e-10, 2.19e-9]	free
δ	1.19 [0.679, 2.23]	1.19 [0.679, 2.23]	1.19 [0.679, 2.23]	shared
$\hat{p}$	8.09e3 [2.71e3, 5.06e4]	4.26e4 [1.41e4, 1.58e5]	3.55e4 [4.04e3, 3.31e5]	free
ε	1.36e-3 [4.27e-5, 0.0499]	1.48e-3 [4.69e-5, 0.0113]	0.189 [6.95e-4, 1.29]	free
c	1.36 [0.771, 2.55]	1.36 [0.771, 2.54]	1.36 [0.770, 2.54]	pooled
α	9.6 [8.96, 9.96]	9.6 [8.96, 9.96]	9.6 [8.96, 9.96]	shared
s_0	2.90e-4 [2.29e-5, 1.10e-3]	2.90e-4 [2.29e-5, 1.10e-3]	2.90e-4 [2.29e-5, 1.10e-3]	shared
d	3.42e-3 [1.73e-3, 6.70e-3]	0.105 [0.0582, 0.178]	0.182 [0.107, 0.300]	free
h	12.3 [6.19, 25.1]	12.2 [6.15, 24.9]	12.5 [6.27, 25.6]	pooled
s_d	2.71e-6 [3.86e-8, 1.16e-4]	3.72e-6 [4.52e-8, 1.69e-4]	3.39e-6 [4.12e-8, 1.51e-4]	pooled
θ	0.0207 [4.83e-4, 1.32]	0.0158 [3.69e-4, 1.20]	0.0171 [3.94e-4, 1.24]	pooled
$\mathrm{cum}F_{50}$	4.44e4 [1.95e4, 5.17e5]	6.49e4 [2.90e4, 7.23e5]	20.5 [5.23, 57.8]	free
V_0	4.01e7 [2.15e7, 7.28e7]	2.01e8 [1.05e8, 3.58e8]	3.28e8 [3.68e7, 9.58e8]	free
D_0	2.61e4 [1.03e3, 3.99e5]	2.96e6 [1.31e5, 4.32e7]	2.58e7 [1.08e6, 3.85e8]	free
σ_V		0.381 [0.324, 0.457]		shared
σ_D		0.513 [0.435, 0.616]		shared
σ_F		30.7 [29.0, 31.3]		shared
T_0		4e6 (same in every group)		fixed

Parameter definitions. β, infection rate (g copies^{-1} day^{-1}); δ, infected-cell loss rate (day^{-1}); $\hat{p}$, progeny-genome production per infected cell (copies cell^{-1} day^{-1}); ε, IFN suppression of viral output (mL pg^{-1}); c, virus clearance rate (day^{-1}); α, IFN decay rate (day^{-1}); s_0, baseline IFN induction (pg mL^{-1} day^{-1} per cell); d, DVG production fraction (dimensionless); h, DVG clearance rate (day^{-1}); s_d, DVG-dependent IFN induction; θ, DVG saturation parameter (inverse DVG load); $\mathrm{cum}F_{50}$, cumulative IFN exposure at half-maximal negative feedback (pg mL^{-1} day); V_0, initial virus (copies g^{-1}); D_0, initial DVG load (copies g^{-1}). σ_V and σ_D are observation-noise scales on the $\log_{10}$ scale; σ_F is the observation-noise scale for IFN on the linear pg mL^{-1} scale. T_0 is the fixed initial target-cell abundance (cells g^{-1}).

bioRxiv preprint doi: https://doi.org/10.64898/2026.10.30.763053; this version posted October 31, 2026. The copyright holder for this preprint (which was not certified by peer review) is the author/funder, who has granted bioRxiv a license to display the preprint in perpetuity. It is made available under a CC-BY 4.0 International license.

Inference details: priors, likelihood, initial conditions, and credible intervals

This section records the prior families, the observation likelihood, the fixed initial conditions, and the credible-interval definition, so the fits can be reproduced from the deposited data alone. Numeric values are the *in vivo* settings; the *in vitro* fits use the same prior families and likelihood with data-scaled hyperparameters.

Priors. Each kinetic rate has a log-normal prior, written $\log(\cdot) \sim \text{Normal}(\mu, \sigma)$ on the log parameter and truncated to the bounds in S2 Table. The DVG fraction uses a logit-normal prior, $\text{logit}(d) \sim \text{Normal}(\mu_d, 3)$, and the initial DVG load D_0 a log-normal prior. The observation-noise scales σ_V and σ_D use Exponential(0.5), and σ_F uses Exponential(0.0319). A weakly-informative log-normal prior on $R_0 = \beta(1-d)\hat{p}T_0/(\delta c)$ (the no-DVG variant drops the $1-d$ factor), centred at $\frac{1}{2}[\log R_{0,\text{lo}} + \log R_{0,\text{hi}}]$ with $R_{0,\text{lo}} = 1$, $R_{0,\text{hi}} = 200$ and scale 2, links β, $\hat{p}$, δ, and c. The parameterisation enforces $c > \delta$ and $h > \delta$.

Likelihood. Virus and DVG enter on the $\log_{10}$ scale, IFN on the linear $\text{pg}\,\text{mL}^{-1}$ scale it is measured on. Virus and DVG use a normal likelihood, $y_V \sim \text{Normal}(\log_{10} V, \sigma_V)$ and $y_D \sim \text{Normal}(\log_{10}(D/m_1), \sigma_D)$, where y_D is the estimated DVG level $\log_{10} D_{\text{obs}}$ and m_1 links it to the model state as $D = m_1 D_{\text{obs}}$. Here $m_1 = 1$: virus is counted as full-length genome copies per gram and DVG as defective genome copies per gram, so the two are already in one unit and no conversion enters. The split of output by d and $1-d$ is then between commensurate quantities, and the values reported for D, D_0 and θ are on that genome scale, in copies g^{-1}. This differs from the earlier fits of this model, which measured virus as PFU g^{-1} and carried $m_1 = 5 \times 10^{-5}$ to put the two on a common particle scale; the constant is retained in the likelihood so the two readings share one expression. IFN uses a normal likelihood on the linear scale, $y_F \sim \text{Normal}(F, \sigma_F)$, with σ_F bounded above by 31.3 $\text{pg}\,\text{mL}^{-1}$, the within-timepoint standard deviation of the measurements themselves. Below-detection observations are left-censored: the contribution is the lower-tail probability of the same distribution evaluated at the detection limit. The limits of detection (LOD) are $\text{LOD}_V = \text{LOD}_D = 0$ on the $\log_{10}$ scale, and $\text{LOD}_F = 12.5$ $\text{pg}\,\text{mL}^{-1}$ on the linear one. Only IFN observations fall below their limit: 14 of 20 in low, 10 of 20 in med and 14 of 20 in high. The IFN limit is the lowest concentration the assay quantifies rather than a figure Penn *et al.* report: they state no detection limit for the lung-homogenate IFN-α ELISA, and the manufacturer specifies the assay over 12.5–400 $\text{pg}\,\text{mL}^{-1}$ [91], the lowest point of its six-point standard curve being 12.5 $\text{pg}\,\text{mL}^{-1}$. Censoring there says of an unquantifiable animal only what the instrument supports, that it lies below the working range. Earlier fits of this model censored at 10^{-2} $\text{pg}\,\text{mL}^{-1}$, three orders below that point, which instead entered such an animal as though it were known to be near zero.

Initial conditions. The target-cell pool starts at $T_0 = 4 \times 10^6$ *in vivo* and 1×10^6 *in vitro*. The main text states the initial infected-cell and IFN states as $I(0) = 0$ and $F(0) = 0$. In the fits these are set to a value very close to zero, so that the solver and the $\log_{10}$ observation model stay defined at $t = 0$; infection is still seeded by V_0 through the βTV term, so the floor carries no biological content. $\text{cum}F(0) = 0$. Initial virus V_0 and (with-DVG) initial DVG load D_0 are fitted (S1 Table).

Credible intervals. Shaded bands are pointwise posterior quantiles: the 50% band is $[25\%, 75\%]$ and the 90% band is $[5\%, 95\%]$. Tabulated intervals are the $[5\%, 95\%]$

bioRxiv preprint doi: https://doi.org/10.64898/2026.10.30.763053; this version posted October 31, 2026. The copyright holder for this preprint (which was not certified by peer review) is the author/funder, who has granted bioRxiv a license to display the preprint in perpetuity. It is made available under a CC-BY 4.0 International license.

Table S2. Prior distributions and bounds for the with-DVG model (*in vivo*). log-Normal (μ, σ) denotes a normal prior on the log parameter, truncated to the bounds shown; logit-Normal denotes a normal prior on logit d, which is unbounded. Bounds give the support of the truncated prior. The no-DVG model drops d, h, s_d, θ, D_0 and σ_D. Group-specific prior means (low / med / high) are listed below the table where they differ; the *in vitro* fits share these families, though not every bound: the tabled numeric bounds are the *in vivo* settings, and the *in vitro* fits rescale some of them to their data (for example the upper bound on β). The blocks separate the four kinds of input. Parameters in the first block take one value across the three groups. Those in the second are free in each group, with one posterior per group. Those in the third are partially pooled: the group values are drawn around a common mean whose spread τ carries its own half-normal prior, so they are neither tied together nor independent. c and h carry their prior on the ratio to δ rather than on the rate itself, which is what makes the orderings $c > \delta$ and $h > \delta$ hold in every group with no rejection step. R_0 is not a free parameter but a function of β, d, $\hat{p}$, T_0, δ and c carrying a soft prior that links them. Rows in the last block are fixed before fitting, take no prior, and are held at the same value in every group, so no posterior is reported for them; T_0 and m_1 are structural choices rather than literature estimates, I_0 and F_0 are numerical floors (see Initial conditions), and the detection limits come from the assays. $m_1 = 1$ here because virus and DVG are both counted as genome copies per gram, so no genome-to-particle conversion enters. Reasons for the two rate orderings are given under Priors.

Parameter	Prior family	(μ, σ)	Bounds or fixed value
One value across the three groups			
δ	log-Normal	(0.235, 2.0)	[0.1, 2.734]
s_0	log-Normal	(−4.61, 3.0)	$[10^{-5}, 10^{3}]$
α	log-Normal	(2.28, 0.05)	[0.1, 10]
Free in each group (one posterior per group)			
β	log-Normal	(−21.5, 6.0)	$[10^{-18}, 10^{-4}]$
$\hat{p}$	log-Normal	(13.2, 6.0)	$[10^{-3}, 10^{12}]$
ε	log-Normal	(−4.60, 3.0)	$[10^{-5}, 10^{3}]$
d	logit-Normal	$(\mu_d, 3.0)$	ordered $d_{\text{low}} < d_{\text{med}} < d_{\text{high}}$
$\text{cum}F_{50}$	log-Normal	(4.61, 3.0)	$[\text{cum}F_{50}^{\text{min}}, 10^{8}]$
V_0	log-Normal	$(\mu_{V_0}, 2.0)$	$[6.48 \times 10^{3}, 8.82 \times 10^{11}]$
D_0	log-Normal	$(\mu_{D_0}, 2.0)$	$[0, D_0^{\text{max}}]$
Partially pooled across groups			
c	log-Normal on $c/\delta - 1$	$(-2.0, 0.2)$; $\tau \sim$ half-N$(0, 0.125)$	$c > \delta$
h	log-Normal on $h/\delta - 1$	$(2.3, 0.2)$; $\tau \sim$ half-N$(0, 0.0938)$	$h > \delta$
θ	log-Normal on the mean	$(-3.91, 3.0)$; $\tau \sim$ half-N$(0, 0.25)$	> 0
s_d	log-Normal on the mean	$(-12.8, 3.0)$; $\tau \sim$ half-N$(0, 0.281)$	> 0
Observation noise and the soft R_0 prior			
σ_V, σ_D	Exponential	rate 0.5	[0, 5]
σ_F	Exponential	rate 0.0319	[0, 31.3]
R_0	log-Normal (soft)	(2.65, 2.0)	—
Fixed inputs — not estimated, no prior, identical across groups			
T_0	fixed	—	4×10^{6} cells g^{-1} (*in vivo*); 1×10^{6} cells well^{-1} (*in vitro*)
m_1	fixed	—	1 (virus and DVG share one unit)
I_0, F_0	fixed	—	a value very close to zero (numerical floor)
$\text{cum}F(0)$	fixed	—	0
LOD_V, LOD_D, LOD_F	fixed	—	0, 0 ($\log_{10}$ copies g^{-1}), 12.5 pg mL^{-1} (linear)

Group-specific prior means (low / med / high): $\mu_d = -4 / -2 / +1$; $\mu_{V_0} = 18.0 / 19.7 / 20.6$; $\mu_{D_0} = 10.3 / 15.2 / 17.2$; $D_0^{\text{max}} = 2.88 \times 10^{7} / 4.12 \times 10^{9} / 3.03 \times 10^{10}$; $\text{cum}F_{50}^{\text{min}} = 1.81 \times 10^{4} / 2.71 \times 10^{4} / 10^{-3}$, the floor that holds the feedback open in low and med.

bioRxiv preprint doi: https://doi.org/10.64898/2026.10.30.763053; this version posted October 31, 2026. The copyright holder for this preprint (which was not certified by peer review) is the author/funder, who has granted bioRxiv a license to display the preprint in perpetuity. It is made available under a CC-BY 4.0 International license.

quantiles. These are quantiles of the posterior, not normal approximations.

Data availability. The processed virus, DVG, and IFN time series fed to every fit, the fixed model inputs (T_0, m_1, F_0, detection limits), and the posterior-median tables for all fits are deposited as plain CSV files in the project repository (`https://github.com/yimei-li/within-host-dip-iav-ode`).

bioRxiv preprint doi: https://doi.org/10.64898/2026.10.30.763053; this version posted October 31, 2026. The copyright holder for this preprint (which was not certified by peer review) is the author/funder, who has granted bioRxiv a license to display the preprint in perpetuity. It is made available under a CC-BY 4.0 International license.

Analytic argument: the IFN decline requires negative feedback

The model with positive IFN response only keeps $\frac{dF}{dt} \geq 0$ while virus and DVGs rise. We first analyse this model, in which IFN follows viral induction (sI) and constant extracellular clearance (αF):

$$\frac{dF}{dt} = s(D)I - \alpha F \tag{5}$$

At 24 hours, IFN reaches its peak in the high-DVG group of the *in vivo* data of Penn *et al.* (Fig 3A), the group this argument is about; in the low- and medium-DVG groups IFN peaks at 48 hours instead. At this maximum, production approximately balances clearance:

$$s_{24\text{h}} I_{24\text{h}} \approx \alpha F_{24\text{h}} \tag{6}$$

An IFN drop at 48 hours requires $\frac{dF}{dt} < 0$. This model keeps $\frac{dF}{dt} \geq 0$:

1. Because $V_{48\text{h}} > V_{24\text{h}}$, the infected cell population ($I_{48\text{h}}$) remains high.
2. The DVG-dependent induction rate is a saturating function: $s(D) = s_0 + s_d D/(1 + \theta D)$. This function is strictly increasing in D, and $D_{48\text{h}} > D_{24\text{h}}$, so $s_{48\text{h}} \geq s_{24\text{h}}$.
3. The total IFN source term at 48 hours is at least as large as it was at 24 hours:
$$(sI)_{48\text{h}} \geq \alpha F_{24\text{h}} \tag{7}$$
4. Meanwhile, the clearance term at 48 hours is much smaller because the absolute IFN concentration has already fallen ($F_{48\text{h}} \ll F_{24\text{h}}$).

Substituting these facts into the model equation gives:

$$\left.\frac{dF}{dt}\right|_{48\text{h}} \geq \alpha F_{24\text{h}} - \alpha F_{48\text{h}} > 0 \tag{8}$$

Constant clearance (α) keeps $\frac{dF}{dt} \geq 0$ while the viral stimulus stays high.

Contradiction: the model with positive IFN response only forces $\frac{dF}{dt} \geq 0$ while the data show an IFN drop. This model couples IFN to the viral trajectory. It keeps $\frac{dF}{dt} \geq 0$ at 48 hours, while the measured IFN in that group drops 8-fold between 24 and 48 hours.

Cumulative-IFN negative feedback resolves this contradiction. As IFN accumulates, the factor $g(\text{cum}F)$ (Eq. 3) lowers the induction source independently of current viral load. Early in infection, $\text{cum}F \ll \text{cum}F_{50}$, so $g(\text{cum}F) \approx 1$ and IFN production proceeds. By 48 hours, the early IFN peak has raised $\text{cum}F$, and $g(\text{cum}F)$ reduces the effective production rate, consistent with negative IFN feedback:

$$s_{48\text{h}}\, I_{48\text{h}} \cdot g(\text{cum}F_{48\text{h}}) < \alpha F_{48\text{h}} \tag{9}$$

The reduction lets the constant clearance term exceed the induction term, so $\frac{dF}{dt}$ becomes negative at the later time. This reproduces the early IFN decline even while virus and DVGs remain abundant.

bioRxiv preprint doi: https://doi.org/10.64898/2026.10.30.763053; this version posted October 31, 2026. The copyright holder for this preprint (which was not certified by peer review) is the author/funder, who has granted bioRxiv a license to display the preprint in perpetuity. It is made available under a CC-BY 4.0 International license.

Fitting virus and IFN alone, without the DVG compartment

Most infection experiments measure virus and IFN but not DVGs, even though DVGs arise in almost any influenza infection. We therefore asked what this model recovers from virus and IFN alone. We refit it without the DVG compartment: the D state and the parameters d, h, D_0, s_d and θ are dropped, the induction term $s(D)\,I$ reduces to $s_0 I$, the virus production term loses its $(1-d)$ factor, and the DVG observations leave the likelihood. Everything else, including the priors, the group sharing and the sampler settings, matches the with-DVG fit. All chains converged, with no divergent transitions.

The cumulative-IFN feedback comes back in the same place and at a similar strength. Its threshold in the high-DVG group is $\mathrm{cum}F_{50} = 30.2$ pg day mL^{-1} (90% credible interval 9.2–82.7), against 20.5 (5.2–57.8) in the with-DVG fit, and the cumulative exposure reaches 3.1 times that threshold by day 4.5, against 4.3 times with DVGs. In both fits the feedback stays open in the low- and medium-DVG groups, where their thresholds carry a floor (S2 Table). So the inoculum that gives the sharp IFN decline is the one that engages the feedback, whether or not the DVG measurements enter the likelihood.

Both structures reproduce the measured virus and IFN time courses. The DVG compartment earns its place by predicting the DVG measurements themselves and by carrying the mechanism to other DVG doses, rather than by fitting virus and IFN better. Posteriors and the fit figure for the no-DVG model are deposited on GitHub.

Applications: classic pony and human-titre datasets revisited (no cumulative-IFN feedback)

Both classical datasets analysed here — the equine pony data of Pawelek *et al.* [34] and the human titre data of Baccam *et al.* [40] — predate the cumulative-IFN-exposure formulation introduced in this paper. We apply the DVG-extended model in its no-cumulative-IFN-feedback variant ($g(\mathrm{cum}F) = 1$). For the pony data [34], IFN measurements are present and the IFN equation is retained as $dF/dt = s(D)\,I - \alpha F$. For the human-titre data [40], IFN measurements are absent and F is omitted entirely. This isolates virus–DIP competition as a candidate mechanism in the absence of an explicit IFN-shutoff term. The double peak in the human titre data would be worth exploring in its own right. Our fits here reproduce the shape but do not try to separate the mechanisms that could produce it.

Pony model (S3 Fig). The pony fits use the DVG-extended model with a DIP-dependent cell-entry term and no cumulative-IFN feedback. States are T, I, V, D, F:

$$\frac{dT}{dt} = -\hat{\beta}\,T\,V, \qquad \hat{\beta} = \frac{\beta\,V}{V + \varepsilon\,D},$$
$$\frac{dI}{dt} = \hat{\beta}\,T\,V - \delta\,I, \qquad p(F) = \frac{\hat{p}}{1 + \varepsilon_2\,F},$$
$$\frac{dV}{dt} = (1-d)\,p(F)\,I - c\,V, \qquad s(D) = s_0 + s_d\,\frac{D}{1 + \theta_s\,D},$$
$$\frac{dD}{dt} = d\,p(F)\,I - h\,D,$$
$$\frac{dF}{dt} = s(D)\,I - \alpha\,F.$$

Here ε is the virus–DIP competition strength at cell entry and ε_2 is IFN suppression of viral output. Because this model carries no genome-to-particle conversion, ε also

bioRxiv preprint doi: https://doi.org/10.64898/2026.10.30.763053; this version posted October 31, 2026. The copyright holder for this preprint (which was not certified by peer review) is the author/funder, who has granted bioRxiv a license to display the preprint in perpetuity. It is made available under a CC-BY 4.0 International license.

absorbs the fraction of defective genomes that are not packaged into particles. Both symbols are local to this equine model: elsewhere in the paper ε denotes IFN suppression of output and no ε_2 appears.

Human-titre model (S4 Fig). The DIP variants of the Baccam fits use a two-stage eclipse (I_1, I_2) with a DIP-dependent cell-entry term and no IFN state (DIP-only). The DIP-only system is

$$\begin{aligned}
\frac{dT}{dt} &= -\beta\, T\, V, \qquad\qquad \beta = \frac{\hat{\beta}}{1+\varepsilon_3\, D},\\
\frac{dI_1}{dt} &= \beta\, T\, V - k\, I_1,\\
\frac{dI_2}{dt} &= k\, I_1 - \delta\, I_2,\\
\frac{dV}{dt} &= (1-d)\, p\, I_2 - c\, V,\\
\frac{dD}{dt} &= d\, p\, I_2 - h\, D,
\end{aligned}$$

with k the eclipse-to-productive transition rate and ε_3 the DIP-load entry-shield strength; initial conditions $I_1(0) = I_2(0) = 0$, $T(0) = T_0$, $V(0) = V_0$, $D(0) = D_0$. The DIP+IFN variant adds the Baccam IFN term.

bioRxiv preprint doi: https://doi.org/10.64898/2026.10.30.763053; this version posted October 31, 2026. The copyright holder for this preprint (which was not certified by peer review) is the author/funder, who has granted bioRxiv a license to display the preprint in perpetuity. It is made available under a CC-BY 4.0 International license.

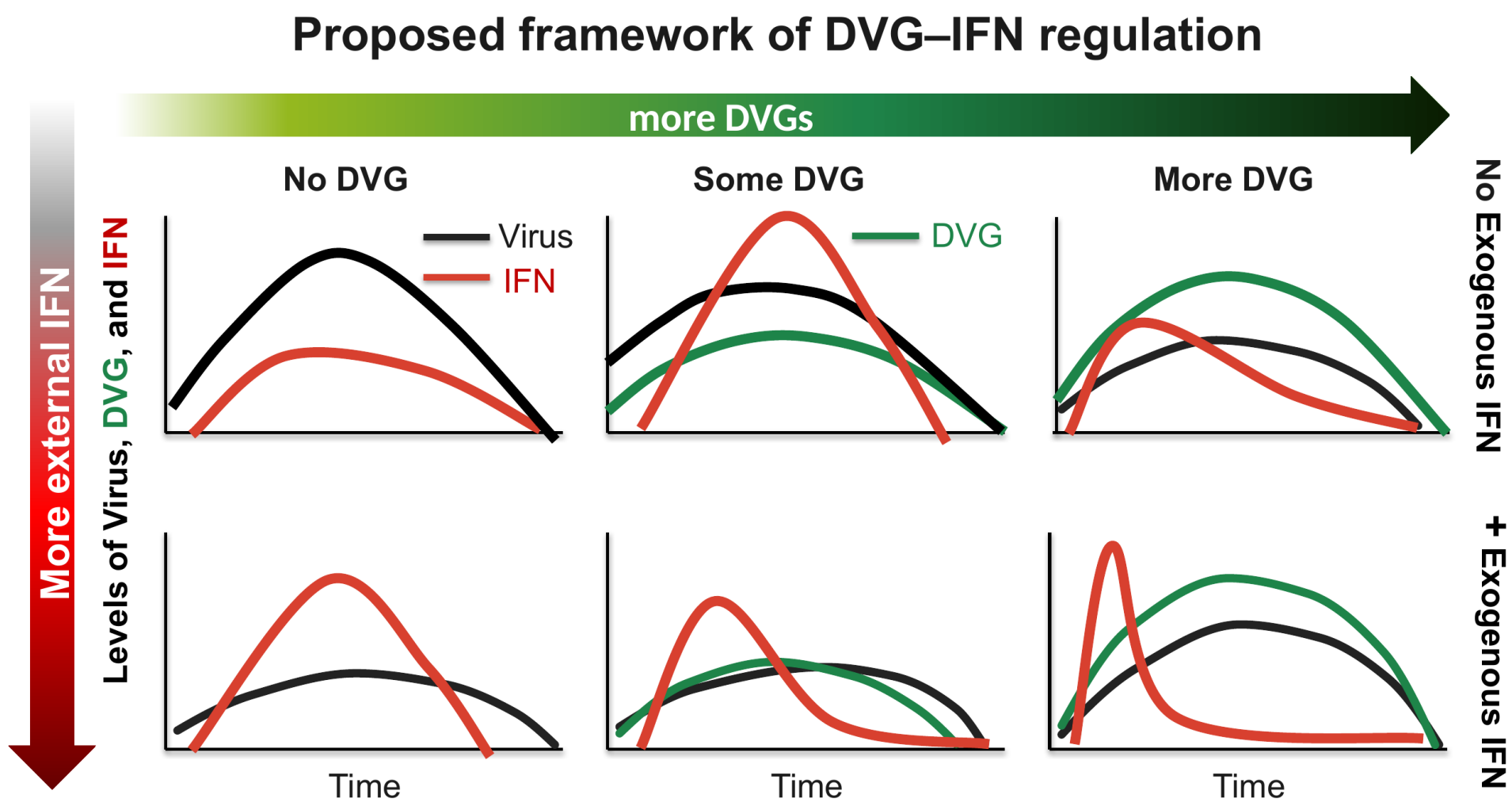

Fig S1. Schematic of virus particle, DVG, and IFN dynamics as a function of DVG and exogenous IFN levels. Columns: no DVG, some DVG, and high DVG levels. Rows: absence (top) and presence (bottom) of exogenous IFN.

bioRxiv preprint doi: https://doi.org/10.64898/2026.10.30.763053; this version posted October 31, 2026. The copyright holder for this preprint (which was not certified by peer review) is the author/funder, who has granted bioRxiv a license to display the preprint in perpetuity. It is made available under a CC-BY 4.0 International license.

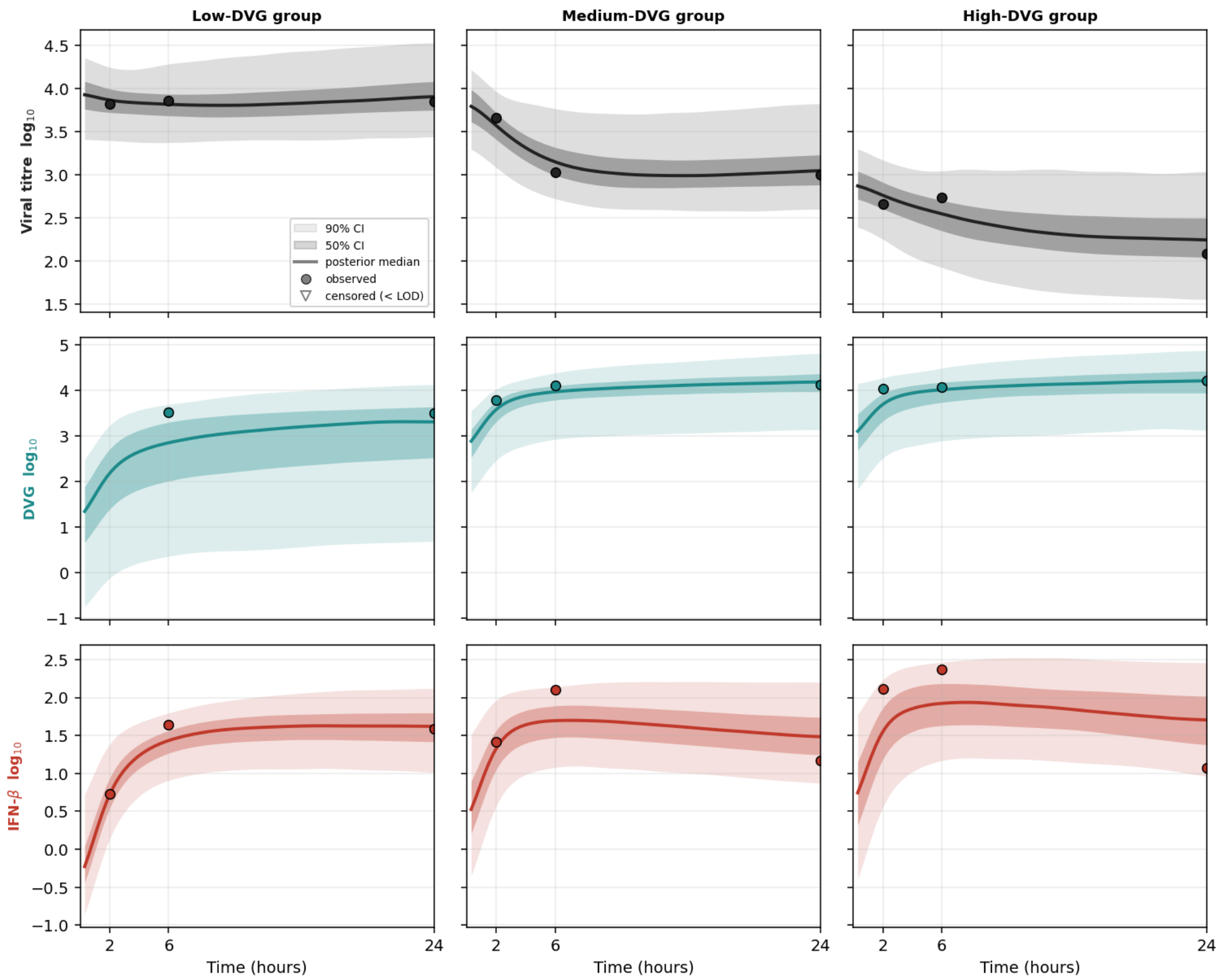

Fig S2. ***In vitro*** **DVG-extended model fits to virus, DVG, and IFN-β mRNA time courses.** Same data and DVG groups as S5 Fig. The DVG-extended model jointly fits virus (PB1 full genome), DVG (PB1 DVGs), and IFN-β mRNA. Rows show virus, DVG, and IFN-β mRNA; columns show the low-, medium-, and high-DVG groups, with one y axis per row.

bioRxiv preprint doi: https://doi.org/10.64898/2026.10.30.763053; this version posted October 31, 2026. The copyright holder for this preprint (which was not certified by peer review) is the author/funder, who has granted bioRxiv a license to display the preprint in perpetuity. It is made available under a CC-BY 4.0 International license.

Earlier or later IFN shutoff changes the host trade-off; no single $\mathrm{cum}F_{50}$ minimises all four costs at once. After fitting the cumulative-IFN half-saturation threshold ($\mathrm{cum}F_{50}$), we asked how host outcome would change if this threshold were higher or lower. For each DVG inoculum group, we held all other parameters at their fitted posterior medians and varied only $\mathrm{cum}F_{50}$: earlier shutoff ($\mathrm{cum}F_{50}/2$), delayed shutoff ($10\,\mathrm{cum}F_{50}$), and feedback removed ($g(\mathrm{cum}F) \equiv 1$). The released case is the dashed curve of Fig 3C. Earlier shutoff lowered the IFN peak and the area under the IFN curve but raised the viral peak and prolonged the high-virus period; delayed shutoff did the reverse, and removing the feedback entirely sat at the far end of this trade-off with the highest IFN and the lowest virus.

The same DVG inoculum group occupied different feedback states depending on $\mathrm{cum}F_{50}$. No single $\mathrm{cum}F_{50}$ minimised all four costs (IFN exposure, virus, infected cells, target cells) simultaneously. The preferred shutoff time depends on which cost matters most.

bioRxiv preprint doi: https://doi.org/10.64898/2026.10.30.763053; this version posted October 31, 2026. The copyright holder for this preprint (which was not certified by peer review) is the author/funder, who has granted bioRxiv a license to display the preprint in perpetuity. It is made available under a CC-BY 4.0 International license.

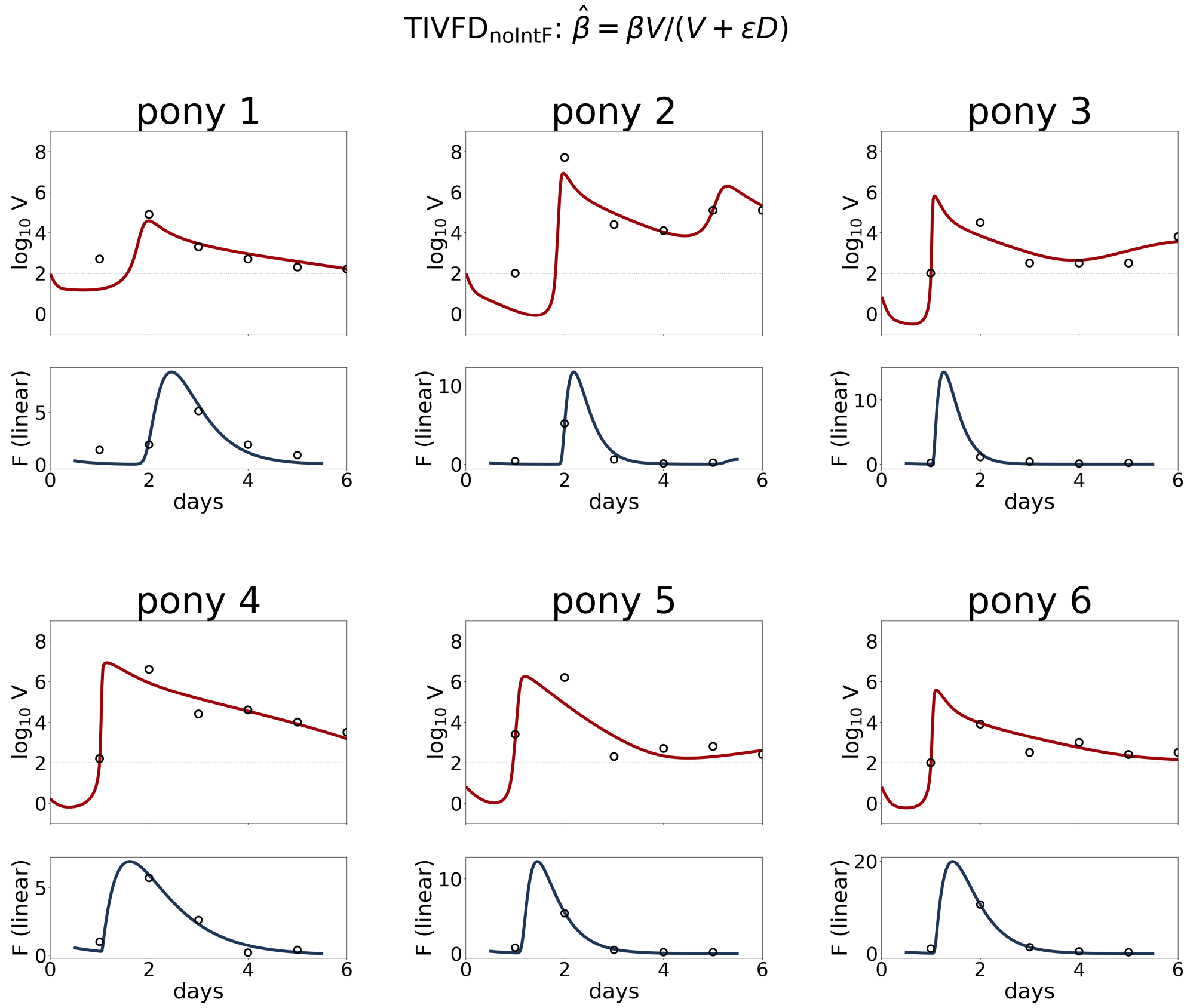

Fig S3. DVG-included fits to the equine influenza pony data. The model was fit to the first 6 days of viral-load and IFN data from the six ponies analysed by Pawelek *et al.* [34]. Each column shows one pony, with viral load on the top row and IFN on the bottom row. Model: the within-host DVG model (Eq 1) with β replaced by a DIP-dependent cell-entry term $\hat{\beta} = \beta V/(V + \varepsilon D)$ and no cumulative-IFN feedback ($dF/dt = s(D)\,I - \alpha F$). Per-pony maximum a posteriori fits; key parameters span $\beta \approx 0.7$–3×10^{-4}, the competition strength $\varepsilon \approx 0.2$–4, and the DVG production fraction $d \approx 0.2$–0.5.

bioRxiv preprint doi: https://doi.org/10.64898/2026.10.30.763053; this version posted October 31, 2026. The copyright holder for this preprint (which was not certified by peer review) is the author/funder, who has granted bioRxiv a license to display the preprint in perpetuity. It is made available under a CC-BY 4.0 International license.

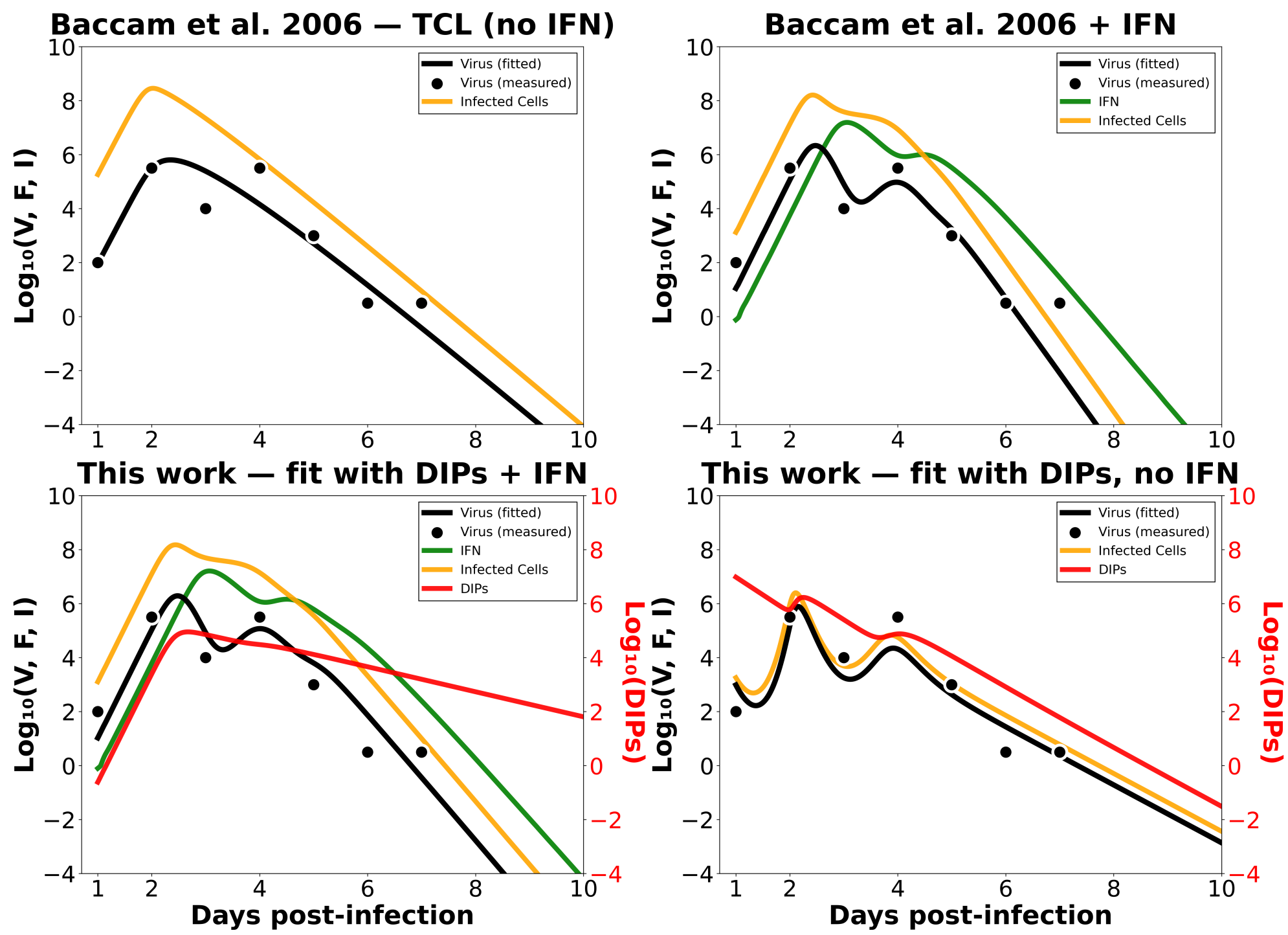

Fig S4. Revisiting classic human influenza viral kinetics with target-cell, IFN, and DIP-based models. The four panels compare model interpretations of the human influenza data analysed by Baccam *et al.* [40]. The target-cell-limited model explains viral decline through target-cell limitation. The Baccam IFN model adds host antiviral control to reproduce non-monotone viral kinetics. The DIP+IFN model includes both host IFN effects and virus–DIP competition. The DIP-only model shows that virus–DIP competition can reproduce the main viral peak and post-peak decline without an explicit IFN variable. The DIP variants use a two-stage eclipse (I_1, I_2) with a DIP-dependent cell-entry term $\beta = \hat{\beta}/(1 + \varepsilon_3 D)$ and output partition $(1 - d)$; the DIP-only variant omits IFN. DIP-only patient-1 fit: $\hat{\beta} \approx 1.5 \times 10^{-4}$, $\varepsilon_3 \approx 9.5 \times 10^{-6}$, $d \approx 0.30$, $p \approx 20$, $c \approx 30$, $\delta \approx 20$ (day^{-1}), $D_0 \approx 292$.

bioRxiv preprint doi: https://doi.org/10.64898/2026.10.30.763053; this version posted October 31, 2026. The copyright holder for this preprint (which was not certified by peer review) is the author/funder, who has granted bioRxiv a license to display the preprint in perpetuity. It is made available under a CC-BY 4.0 International license.

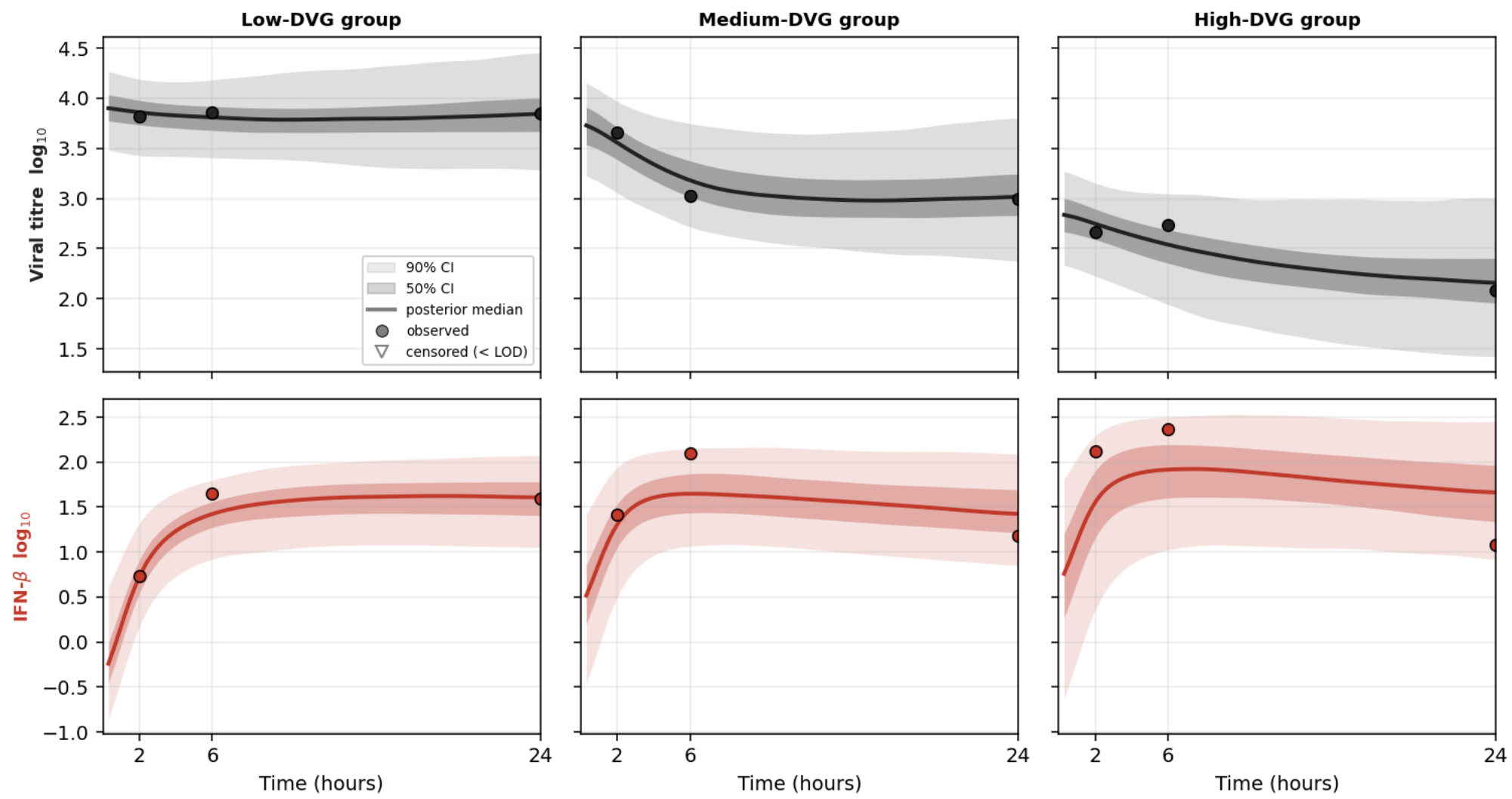

Fig S5. *In vitro* no-DVG model fits to virus and IFN-β mRNA time courses. Penn *et al.* [20] PB1 full-genome and IFN-β mRNA data, grouped by DVG group (low, med, high). Lines: posterior median. Shaded bands: pointwise posterior quantile intervals (50%: 25%–75%; 90%: 5%–95%). Points: observed data. The no-DVG model fits V and F only; it does not track DVGs. Rows show virus and IFN-β mRNA; columns show the low-, medium-, and high-DVG groups, with one y axis per row.

bioRxiv preprint doi: https://doi.org/10.64898/2026.10.30.763053; this version posted October 31, 2026. The copyright holder for this preprint (which was not certified by peer review) is the author/funder, who has granted bioRxiv a license to display the preprint in perpetuity. It is made available under a CC-BY 4.0 International license.

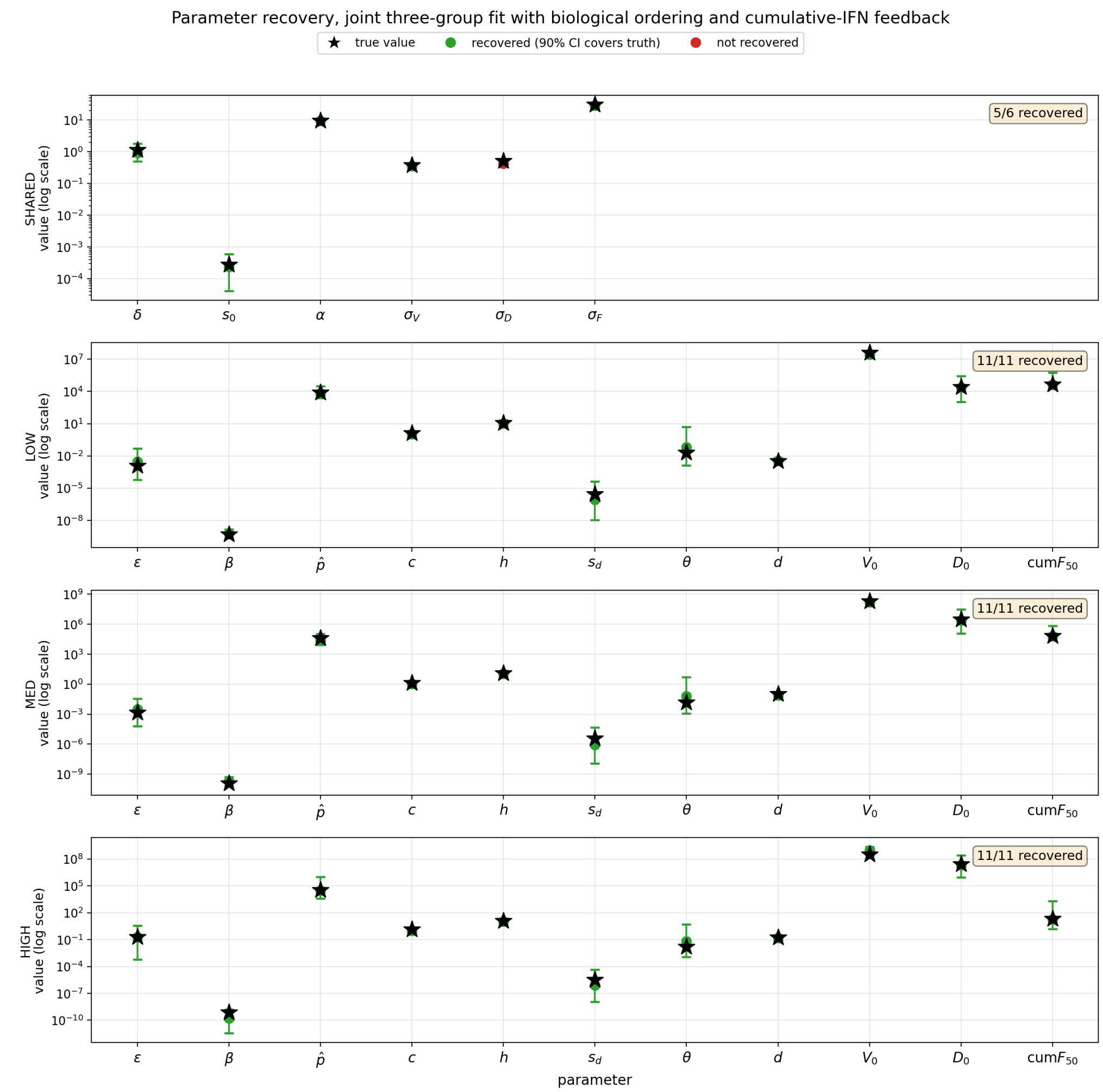

Fig S6. Simulation-based parameter recovery for the with-DVG, cumulative-IFN-feedback model. For each inoculum group (LOW, MED, HIGH), the posterior median of every fitted parameter (S1 Table) was taken as ground truth (black stars). The six-state ODE (Eq 1) was forward-simulated at these values at the Penn *et al.* [20] observation times, and Stan-likelihood-matched noise was added: V and D Normal on the $\log_{10}$ scale, F Normal on the linear $\mathrm{pg\,mL^{-1}}$ scale it is fitted on. The same Stan model was then refit to the synthetic data under the same priors, bounds, and NUTS settings. Green dots with bars give the posterior median and 90% credible interval of each refit parameter; a parameter counts as recovered when its 90% interval covers the truth. The fit has 39 estimated quantities: six held to one value across the groups (δ, s_0, α, σ_V, σ_D, σ_F) and eleven free or partially pooled in each of the three. Thirty-eight of the 39 are recovered, including every rate, the defective-genome fraction d and the threshold $\mathrm{cum}F_{50}$ in all three groups. The remaining one is the DVG observation noise σ_D (red dot), whose truth of 0.514 lies 2% above the upper end of its interval, 0.503.

bioRxiv preprint doi: https://doi.org/10.64898/2026.10.30.763053; this version posted October 31, 2026. The copyright holder for this preprint (which was not certified by peer review) is the author/funder, who has granted bioRxiv a license to display the preprint in perpetuity. It is made available under a CC-BY 4.0 International license.

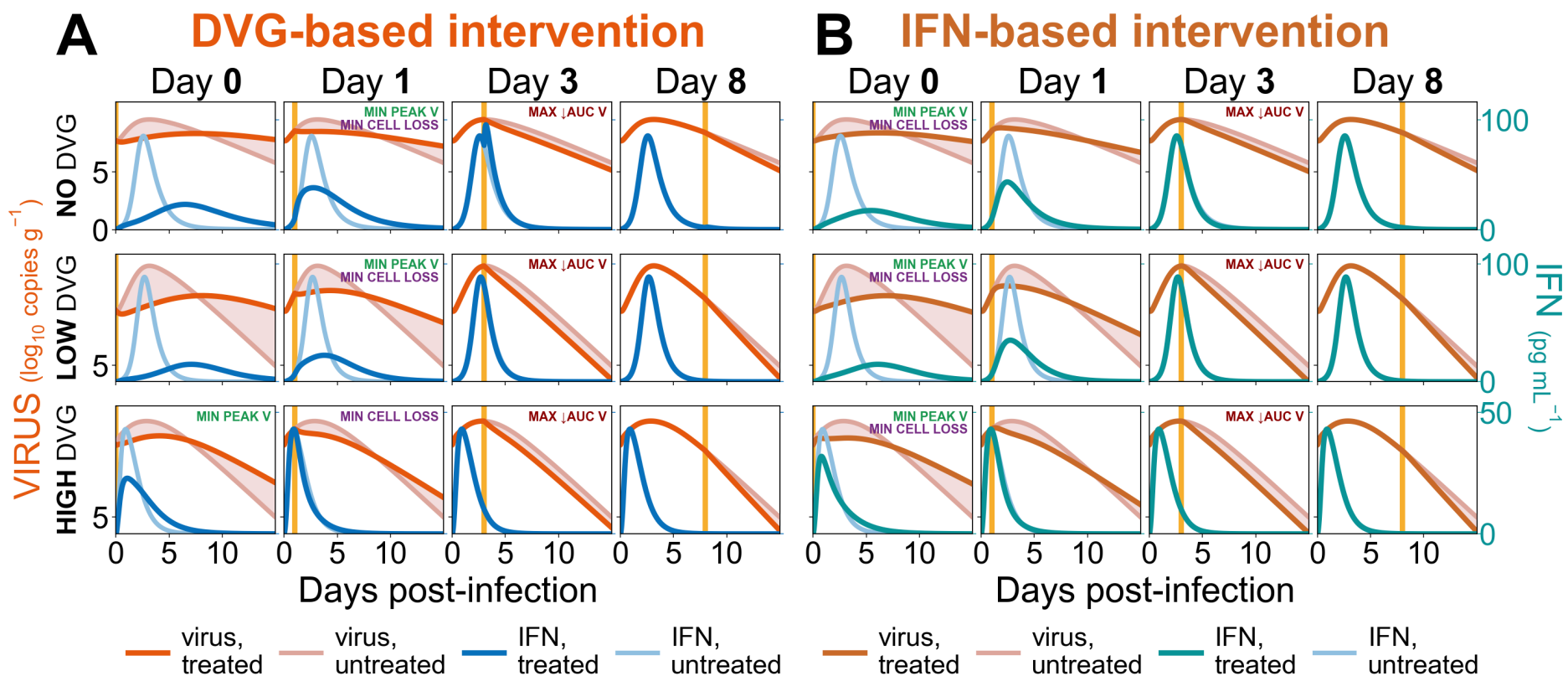

Fig S7. Simulated DVG- and IFN-based interventions across initial DVG levels and treatment times. **(A)** DVG-based intervention and **(B)** IFN-based intervention initiated on days 0, 1, 3, or 8 (yellow lines). Rows show the no-, low-, and high-DVG conditions. Virus and IFN trajectories are shown for treated and untreated conditions; shading indicates the difference in viral trajectories. Curves show the rank-trimmed mean across 200 posterior draws. MIN V indicates the treatment time producing the largest reduction in viral burden within each DVG condition.
